# Evaluation of Antibiotic Resistance and Virulence Genes among Clinical Isolates of *Pseudomonas aeruginosa *from Cancer Patients

**DOI:** 10.31557/APJCP.2020.21.5.1333

**Published:** 2020-05

**Authors:** Naveed Ahmed, Zeshan Ali, Mahpara Riaz, Basit Zeshan, Javed Iqbal Wattoo, Muhammad Naveed Aslam

**Affiliations:** 1 *Department of Microbiology, University of Central Punjab Lahore, Pakistan. *; 2 *Department of Microbiology, Pakistan Kidney and Liver Institute and Research Center, Lahore, Pakistan.*; 3 *College of Food Engineering and Nutritional Sciences, Shaanxi Normal University. Xi’an 710119, China. *

**Keywords:** P. aeruginosa, virulent genes, antibiotic resistance, chemotherapy, phenotypic characterization

## Abstract

**Objectives:**

The objectives of this study were to evaluate *P. Aeruginosa* isolates from cancer patients for the phenotypic pattern of antibiotic resistance and to detect the gene responsible for virulence as well as antibiotic resistance.

**Methods:**

A total of 227 *P. aeruginosa* isolates were studied and 11 antibiotics were applied for susceptibility testing. PCR detection of the genes BIC, TEM, IMP, SPM, AIM, KPC, NDM, GIM, VIM, OXA, toxA and oprI was done. Finally, the carbapenem resistant isolates were tested for phenotypic identification of carbapenemase enzyme by Modified Hodge test.

**Results:**

The results showed that the isolates were resistant to imipenem (95%), cefipime (93%), meropenem (90%), polymixin B (71%), gentamicin (65%), ciprofloxacin (48%), ceftazidime (40%), levofloxacin (39%), amikacin (32%), tobramycin (28%) and tazobactum (24%). The PCR detection of the carbapenem resistant genes showed 51% isolates were positive for IMP, GIM and VIM, 38% for AIM and SPM, 30% for BIC, 20% for TEM and NDM, 17% for KPC and 15% for OXA. However, toxA and oprI genes were not detected. 154 carbapenem resistant isolates were found positive phenotypically for carbapenemase enzyme identification by Modified Hodge test.

**Conclusion:**

The co-existence of multiple drug-resistant bodies and virulent genes has important implications for the treatment of patients. This study provides information about treating drug-resistant *P. Aeruginosa* and the relationship of virulent genes with phenotypic resistance patterns.

## Introduction

Drug resistance causes major problems in patients suffering from cancer or admitted to intensive care units which causes so many complications in the treatment (Caselli et al., 2010). Cancer patients are at high risk of developing antibiotic resistance mainly in patients with leukemia, breast cancer, lung cancer and GIT cancer. The state of bacteremia in immune compromised patients or the patients who are suffering from leukemia is very dangerous Due to the use of invasive devices, cancer patients undergoing chemotherapy are at high risk of getting infection with multi drug resistant (MDR) Pseudomonas aeruginosa (*P. aeruginosa*) (Lister et al., 2009). Bacteremia due to the MDR *P. aeruginosa* is life threatening infection in cancer patients. The low permeability of its cell wall with mutation leading to the resistance via efflux pump, decreased level of porins plays a major role in development of problem in antibiotic therapy (Ullah et al., 2009). The prevalence of drug resistance in cancer was also increased (Pai et al., 2001).

Multi drug resistant (MDR) strains are responsible to hospital acquired infections, especially in the cancer patients who are at high risk. MDR *P. aeruginosa* causes the major infections in cancer and cystic fibrosis patients (Cornaglia et al., 2011). Because of MDR bacterial strains, the cancer may develop resistance against the chemotherapeutic drugs as well and it is the most important factor playing an important role in the failure of chemotherapy. The patients of hematological malignancies are always on at risk to be infected with *P. aeruginosa*. MDR *P. aeruginosa* is also involved in neutropenia or lymphocyte dysfunctions (Poirel et al., 2011a; de la Fuente-Núñez et al., 2015). Generally, it shows resistance against the β-lactam antibiotics due to high production of cephalosporin AmpC with the mutation in OprD. *P. aeruginosa* producing the metallo-β-lactamase (MBL) was isolated from the breast cancer patient with VIM-7 gene (Lee et al., 2003; Poirel et al., 2011b; Tseng et al., 2013). 

Carbapenems including imipenem and meropenem, have a high and effective anti pseudomonal activity and these have been used as drug of choice for *P. aeruginosa* infections (Picao et al., 2009). The entry of carbapenem in market opened the door for the cure of severe infection due to beta-lactam resistance in microorganism. The clinically significant type of carbapenemases are VIM, IMP and SPM which are encoded by bla (VIM), bla (IMP) and bla (SPM) respectively. Fourteen types of VIMs and twenty three types of IMPs have been identified yet (Lee et al., 2003; Toval et al., 2015). The resistance against carbapenem was 60% in many hospitals and it was mainly due to the production of MBL (Tsakris et al., 2000; Wolter et al., 2008). 

## Materials and Methods

*Sample Collection, Isolation and Identification*

Blood, sputum, urine and pus samples were collected from cancer patients admitted in a tertiary care hospital. After collection, the samples were immediately transported to the Department of Microbiology, University of Central Punjab, Lahore. Isolation and identification was done on the basis of bacterial colony type, colony morphology and macroscopic characteristics followed by Gram’s staining and biochemical tests. *P. aeruginosa* isolates showed positive reactions for citrate and oxidase tests while negative reaction for indole, methyl red, voges-proskauer, urease, H2S reactions and lactose, sucrose and glucose fermentation.

*Antimicrobial Susceptibility Testing*

Following isolation and identification of *P. aeruginosa*, antibiotic susceptibility testing was performed by the disk diffusion method according to the protocol in the CLSI guidelines (2017). Inoculum density was standardized using McFarland standards. The antibiotics used for this study were as follows; amikacin (AK), cefipime (FEP), ceftazidime (CAZ), ciprofloxacin (CIP), gentamicin (CN), imipenem (IPM), levofloxacin (LEV), meropenem (MEM), polymixin B (PB), tobramycin (TOB), and tazobactum (TZP).

*Phenotypic Identification of Carbapenemase Producing Isolates*

Phenotypic characterization of carbapenemase producing isolates was done by Modified Hodge Test as per CLSI guidelines as described by Lee et al., 2003. Briefly, E. coli ATCC 25922 was cultured on MacConkey agar and 0.5 McFarland standard was prepared in 2 mL of normal saline. A 10-µg meropenem disk was placed in the centre of the test area on Muller Hinton (MH) agar. In a straight line, test organism was streaked from the edge of the disk to the edge of the plate and were incubated at 37˚C. After 24 hours of incubation, isolates were proceeded for DNA extraction and molecular detection of genes by PCR.

*Methods for DNA Extraction and PCR Amplification*

DNA Extraction was done using CTAB method. PCR was performed on all the extracted DNA of isolates using the annealing temperature given in (Table 1). The expected sizes of PCR products for the two sets of primers were ranged from 163 to 798 base-pairs (bp). The PCR mixture was incubated for ﬁve minute at 95°C as an initial denaturation step. Initial DNA release and denaturation was at 94^o^C for 30 seconds, annealing temperature for each gene is given in Table 1 and extension was completed at 72^o^C for 50 seconds, followed by 36 repeated cycles and a single, ﬁnal, elongation step at 72°C for 10 minutes (Kateete et al., 2016). The samples were loaded on 1.5% agarose gel and the DNA bands were visualized using gel documentation apparatus (Anjum and Mir, 2010).

## Results

*Sample Collection, Isolation and Identification*

From a total of 410 clinical samples, blood (150), sputum (150), urine (60) and pus (50) samples were collected. On the basis of bacterial culture, 304 samples were positive for different bacteria. Out of these 304 positive bacterial cultures, 227 *P. aeruginosa* were isolated and identified.

*Prevalence of P. aeruginosa on the basis of cancer type*

Prevalence of *P. aeruginosa* on the basis of cancer type showed different variation (Table 2). The presence of MDR *P. aeruginosa* was found all the types of cancers in children and adults undergoing chemotherapy treatment. In children, high prevalence of *P. aeruginosa* was found in patients suffering from acute leukemia while in adults *P. aeruginosa* was found prevalent in patients suffering from breast cancer.

*Antimicrobial Susceptibility Testing*

*P. aeruginosa* showed antibiotic resistance to amikacin (AK) 32%, cefipime (FEP) 93%, ceftazidime (CAZ) 40%, ciprofloxacin (CIP) 48%, gentamicin (CN) 65%, imipenem (IPM) 95%, levofloxacin (LEV) 39%, meropenem (MEM) 90%, polymixin B (PB) 71%, tobramycin (TOB)28%, and tazobactum (TZP) 24% as shown in Table 3.

*Detection of beta lactamase (bla) genes*

**Figure 1:**
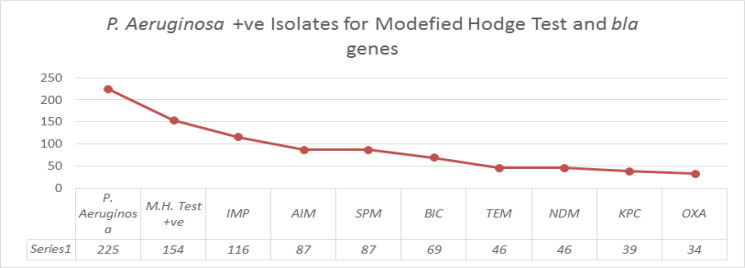
Prevalence of Modified Hodge test +ve and bla Genes in the *P. aeruginosa* Isolates

**Figure 2.**
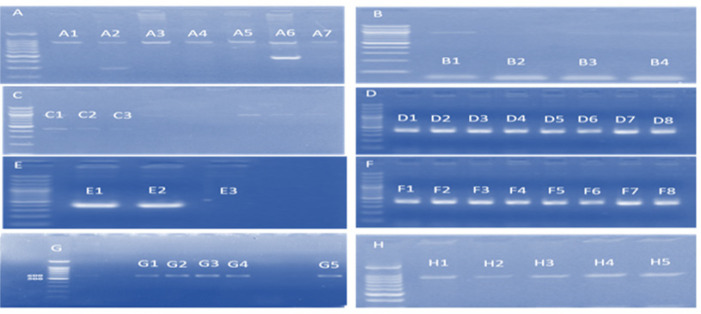
After PCR Gel Electrophoresis Picture Showing Various Bands of +ve Virulence Genes. A-H, 100bp DNA Ladder; A1-A7, KPC; B1 and B4, SPM; C1 and C2, TEM; C3, AIM; D1-D8, IMP; E1-E3, GIM; F1-F8, BIC; G1-4, OXA; G5, VIM; H1-H5, NDM

The PCR detection of the carbapenem resistant genes showed 51% (116) isolates were positive for the IMP, GIM and VIM, 38% (87) for AIM and SPM respectively while BIC gene was found in 30% (69) of isolates. TEM and NDM were detected in 20% (46), KPC was present in 17% (39) isolates and OXA was detected in 15% (34) only. However, toxA and oprI were not found in these isolates (Figure 1). The amplified PCR products of various genes are shown in Figure 2.

*Phenotypic Characterization of Carbapenemase Among Isolates*

Carbapenem resistant samples (n=202) were preceded for phenotypic identification of carbapenemase enzyme and out of which 154 samples were Hodge test positive.

**Table 1:** Nucleotide Sequence of Primer Sets Used for Amplification by PCR

Genes	Primer Sequences (5’-3’)	Annealing temperature (°C)	Product Size (bp)
*BIC*	F: TATGCAGCTCCTTTAAGGGC	60	508
	R: TCATTGGCGGTGCCGTACAC		
*GIM*	F: TCGACACACCTTGGTCTGAA	62	432
	R: TCATTGGCGGTGCCGTACAC		
*TEM*	F: TCGGGGAAATGTGCG	58	440
	R: TGCTTAATCAGTGA GGCACC		
*IMP*	F:GAAGGCGTTTATGTTCATAC	55	587
	R: GTAAGTTTCAAGAGTGATGC		
*AIM*	F:CTGAAGGTGTACGGAAACAC	54	445
	R:GTTCGGCCACCTCGAATTG		
*SPM*	F:TATGCAGCTCCTTTAAGGGC	56	271
	R:ACATTATCCGCTGGAACAGG		
*VIM*	F: GATGGTGTTTGGTCGCATA	52	401
	R: CGAATGCGCAGCACCAG		
*KPC*	F: CGTCTAGTTCTGCTGTCTTG	52	798
	R: CTTGTCATCCTTGTTAGGCG		
*NDM*	F: GGTTTGGCGATCTGGTTTTC	52	621
	R: CGGAATGGCTCATCACGATC		
*OXA*	F: GCGTGGTTAAGGATGAACAC	52	438
	R: CATCAAGTTCAACCCAACCG		

**Table 2:** Prevalence of *P. aeruginosa* in Various Types of Cancer Patients Undergoing Chemotherapy

Diseases/ Cancer Types	Positive Sample (n)	Prevalence %
Acute leukemia (ALL)	61	26.87
Chronic leukemia (CLL)	48	21.14
Acute Myeloid leukemia (AML)	31	13.65
Osteo Sarcoma (OS)	14	6.16
Ewing's sarcoma	7	3.08
Hodgkin's lymphoma	12	5.28
Non-Hodgkin’s lymphoma	9	3.96
Lung Cancer	9	3.96
Breast Cancer	11	4.84
Liver Cancer	13	5.72
Blood Cancer	12	5.28

**Table 3:** Resistance Pattern of *P. aeruginosa *Isolates against Different Antibiotics

	Male		Female			Resistance %
Antibiotics	Sensitive	Resistant	Sensitive	Resistant	Total	
Amikacin (AK)	101	51	52	22	227	32
Cefipime (FEP)	13	139	3	72	227	93
Ceftazidime (CAZ)	90	62	46	29	227	40
Ciprofloxacin (CIP)	74	78	42	33	227	48
Levofloxacin (LEV)	93	59	45	30	227	39
Polymixin B (PB)	34	118	32	43	227	71
Tobramycin (TOB)	106	46	56	19	227	28
Gentamycin (CN)	55	97	23	52	227	65
Tazobactum (TZB)	124	38	57	18	227	24
Imipenem (IPM)	8	144	4	71	227	95
Meropenem (MEM)	11	141	13	62	227	90

## Discussion

In intensive care units of hospitals, *P. aeruginosa* is most common pathogen involved in various infections among immuno-compromised patients. *P. aeruginosa* has developed more sophisticated resistant mechanism against the various antibiotics (Yan et al., 2001). The aim of this study was to isolate, identify the multi drug resistant *P. aeruginosa* and to detect carbapenem resistant genes from clinical samples of cancer patients.

The prevalence of *P. aeruginosa* in this study was 54%, which showed that it may potentially be significant in causing infection among cancer patients. The highest prevalence of *P. aeruginosa* was found in blood sample (50%) as comparison to urine sample (20%) and the prevalence of nasal sample was 30% and the high prevalence relates to the results obtained in previous study (Pagani et al., 2004). The high prevalence in blood sample indicated that it poses high risk in causing bacteremia in cancer patients. The intermediate prevalence of *P. aeruginosa* in nasal sample indicated that it may be transmitted through the physical contact (Caselli et al., 2010; Heltshe et al., 2014). The low prevalence in urine samples showed lesser urinary tract infection in cancer patients. *P. aeruginosa* was found resistant to the majority of detergents and disinfectants (Dawra et al., 2017). The patients with low immunity and with compromised host defense are at high risk of infection. The cross contamination with the hospital isolates played a significant role in causing nosocomial infections (Hammami et al., 2011). The prevalence of Pseudomonas in men was 34% while in women was 46% while in children was 20%. This study revealed that the prevalence in males was higher than in females. In the antibiotic susceptibility pattern it was found that *P. aeruginosa* showing the maximum resistant to all commercially available antibiotics which could be linked to acquired resistant genes in these isolates.

The previous study revealed that the use of broad spectrum antibiotics played a most important role in the development of resistance against antibiotics. There is a need to develop the antimicrobial and disinfectant which act on a biofilm formation and inhibit the biofilm formation (Al-Charrakh et al., 2016). In our study, 32% isolates were resistant to amikacin (AK), 93% to cefipime (FEP), 40% to ceftazidime (CAZ), 48% to ciprofloxacin (CIP), 24% tazobactum (TZP), 71% to polymixin B (PB), 28% to tobramycin (TOB), 65% to gentamicin (CN) and 39% to levofloxacin (LEV). While a study conducted in Mexico showed 61% resistance to amikacin, 63% to cefipime, 68% to ceftazidime, 68% ciprofloxacin, 23% to tazobactum, 67% to polymixin B, 71% resistance to tobramycin. (Toval et al., 2015). A study conducted in Saudi Arabia shows 20% resistance to amikacin, 40% to cefipime, 45% to ceftazidime, 50.3% to ciprofloxacin, 5.42% tazobactum and 40% resistance polymixin B (Al-Agamy et al., 2011). A study conducted in Spain shows 19%, 52%, 53%, 24%, 44% and 59% (Gutierrez et al., 2007), while a study from Egypt shows 15%, 98%, 91%, 56%, 94%, 2.5%, 50% and 43% (Mahmoud et al., 2013). 49% of isolates were found resistant to gentamicin in a previous study conducted in Iran (Fallah et al., 2013).

One of the study, Mahmoud et al., (2013) showed that the *P. aeruginosa* strains were 33.3% resistant to imipenem. In a previous study conducted by Al-Agamy and his colleagues in Iran, 83% isolates were resistant to imipenem (Fallah et al., 2013), Heltshe et al., (2014) from Spain showed 95% resistance rate (Gutierrez et al., 2007). Among gram negative bacteria the *P. aeruginosa* and Acinetobacter showed the high level of resistance against the imipenem which was 37.03% in a study conducted at Egypt (Mahmoud et al., 2013). Ashour and El-Sharif (Ashour and El-Sharif, 2009) concluded that Acinetobacter and Pseudomonas species exhibited the highest resistance levels to imipenem (37.03%) among other Gram-negative organisms. In middle-east, imipenem resistance was shown to be increased while, resistance against the imipenem and meropenem (MEM) in Saudi Arabia was 38.57% & 38% respectively as reported in 2011 (Al-Agamy et al., 2011) and 68% in Spain (Gutierrez et al., 2007). 

According to the European surveillance system in six different European countries the carbapenem resistance was reported about 25%. The resistance against the carbapenem reported in Greece was 51% (Souli et al., 2008). The Bacteria has different type of enzyme which plays an important role in the resistance. This rate of carbapenem resistance reflects a threat limiting the treatment options in our hospitals. This can be explained in part by the increase in consumption of antimicrobial agents in the last decade leading to a selective pressure of antibiotics on *P. aeruginosa* and consequently the bacteria modify the resistant mechanisms (Mahmoud et al., 2013). A similar high rate of resistance has been reported in many other developing countries (Picao et al., 2009). 

Modified Hodge Test (MHT) was an easy and simple test to be performed to detect carbapenemases producing bacteria. There were six types of carbapenemase enzymes IMP, VIM, OXA, KPC, CME and NDM. Modified Hodge Test is non-specific test for the detection of carbapenemases, the results of that test showed the presence of carbapenemase enzyme but type of enzyme responsible for resistance was not conformed. A study conducted by Delphine et al., (2011) showed that MHT results of among 35 carbapenem resistant isolates 24 were MHT positive and MHT positive isolates were genetically conformed for blaIMP by PCR, only two samples were blaIMP positive further confirmation for carbapenemase KPC, VIM and NDM by PCR was done and results showed that 7 samples were KPC positive 1 sample was VIM positive and 3 samples were NDM positive (Girlich et al., 2011).

The most commonly reported families were IMP which was firstly isolated in Japan. A study by Laupland et al., (2005) evaluated the presence of blaIMP gene between 98 MBL producing *P. aeruginosa* isolates in Calgary Health Region in Canada between May 2002 and April 2004 and showed that 19% of them were IMP positive (Laupland et al., 2005). Another study conducted by AL-Kadhmi et al., (2016) showed that 1.3%. prevalence of blaIMP in wounds sample isolated from burn canter (AL-Kadhmi et al., 2016). It showed that the spread patterns of different MBLs between countries were different and their relationships with geographical areas, hygienic conditions and chromosomal structure of bacterial strains should, therefore, be evaluated. MBL mediated imipenem resistance in *P. aeruginosa* has raised many concern in the treatment of infected patients. In 6% isolates were positive for bla (IMP) gene in Iran (Fallah et al., 2013), 20% in Spain (Gutierrez et al., 2007), while in current study IMP was the most commonly detectable gene among *P. aeruginosa*. The prevalence of IMP gene among *P. aeruginosa* was 51%. Previous studies demonstrate that the IMP gene prevalence is the greatest clinical threat (Tseng et al., 2013). IMP and VIM producing *P. aeruginosa* are reported worldwide in different areas (Pai et al., 2001; Lee et al., 2003; Souli et al., 2008).

SPM and AIM genes were firstly isolated from Brazil. In Present study 38% of total isolates were positive for AIM and SMP genes. The result of previous studies supports the findings of our research. In our study 30% (69) isolates were positive with BIC genes, 20% (46) for TEM, NDM. OXA was detected in 15% (34) samples only while in Egypt its prevalence rate was 41.7% (Zafer et al., 2014). KPC was rarely detected in *P. aeruginosa*; however the number of reports of KPC producing *P. aeruginosa* is increasing (Zafer et al., 2014). KPC was isolated in 17% (39) samples, toxA and oprI was not found in isolates.

In conclusion, this study provides information for treating drug resistant *P. aeruginosa* as well as the relationship of virulent genes with phenotypic resistance patterns. In the present study, the high resistance was reported in *P. aeruginosa* isolates. The prevalence of resistant genes IMP, GIM, VIM, SPM, AIM, BIC in *P. aeruginosa* was also increased in isolates from different cancer patients. These findings could be considered beneficial in understanding the mechanism involved in development of antibiotic resistance in *P. aeruginosa* isolates and their relationship with beta-lactamase genes.
